# Hemispheric Asymmetries in Speech Perception: Sense, Nonsense and Modulations

**DOI:** 10.1371/journal.pone.0024672

**Published:** 2011-09-30

**Authors:** Stuart Rosen, Richard J. S. Wise, Shabneet Chadha, Eleanor-Jayne Conway, Sophie K. Scott

**Affiliations:** 1 University College London Speech, Hearing and Phonetic Sciences, University College London Division of Psychology and Language Sciences, London, United Kingdom; 2 Division of Neurosciences and Mental Health, Imperial College London, London, United Kingdom; 3 University College London Institute of Cognitive Neuroscience, University College London Division of Psychology and Language Sciences, London, United Kingdom; The University of Western Australia, Australia

## Abstract

**Background:**

The well-established left hemisphere specialisation for language processing has long been claimed to be based on a low-level auditory specialization for specific acoustic features in speech, particularly regarding ‘rapid temporal processing’.

**Methodology:**

A novel analysis/synthesis technique was used to construct a variety of sounds based on simple sentences which could be manipulated in spectro-temporal complexity, and whether they were intelligible or not. All sounds consisted of two noise-excited spectral prominences (based on the lower two formants in the original speech) which could be static or varying in frequency and/or amplitude independently. Dynamically varying both acoustic features based on the same sentence led to intelligible speech but when either or both acoustic features were static, the stimuli were not intelligible. Using the frequency dynamics from one sentence with the amplitude dynamics of another led to unintelligible sounds of comparable spectro-temporal complexity to the intelligible ones. Positron emission tomography (PET) was used to compare which brain regions were active when participants listened to the different sounds.

**Conclusions:**

Neural activity to spectral and amplitude modulations sufficient to support speech intelligibility (without actually being intelligible) was seen bilaterally, with a right temporal lobe dominance. A left dominant response was seen only to intelligible sounds. It thus appears that the left hemisphere specialisation for speech is based on the linguistic properties of utterances, not on particular acoustic features.

## Introduction

Hemispheric asymmetries in speech and language processing have been linked to differential sensitivities in the left and right auditory cortices for low level acoustic features for more than 50 years [1]. More specifically, the left auditory cortex has been claimed to be specialised for rapid temporal processing and the right for spectral processing [2], [3] especially concerning pitch [4]. It has been similarly suggested that the left auditory cortex samples information over shorter temporal windows than the right, making it more sensitive to rapid acoustic change [5], [6]. While all of these studies were addressing relative rather than absolute differences between the left and right hemispheres, it is notable that the left temporal lobe responses were always either equivalent for the temporal and spectral changes [3] or greater for spectral detail [2]. Likewise the left temporal lobe does not respond selectively to short temporal intervals [6]. It is also notable that no functional imaging study in which basic low-level signal properties are manipulated has revealed a greater activation in the left temporal lobe for different types of acoustic structure, or the rate at which they change. Thus studies of harmonic structure [7], amplitude modulation [8], [9], frequency modulation [9], pitch and melody [10], spectral modulations [11], spectral envelope [12], dynamic spectral ripples [13], increasing rates of click trains [14] and variations in the degree of spectral correlation across time [15] have shown clear bilateral (or even right-biased) activation.

Although incorporating acoustic structure that is more or less similar to that found in speech, such signals are still very limited as direct analogues of speech. No single acoustic cue underpins the perception of speech, with a mix of properties typically utilized by a listener even when making a simple phonetic contrast [16]. Even so, it is clear that intelligibility requires, minimally, information about the spectral dynamics conferred by the peaks in energy (formants), changing in frequency, which arise from the resonances created by the vocal tract [17]. Such moving bands of energy create, of course, modulations in amplitude within the restricted frequency channels that much of the auditory pathway is organized around. Strikingly, only relatively slow modulations are necessary to support the intelligibility of speech, in the region of 16 Hz and below [18], [19].

The central importance of slowly changing spectral information for speech intelligibility is at odds with claims that the left temporal lobe is specialised for rapid temporal processing, if we accept that the left temporal lobe dominates in speech perception [20]. Indeed, a recent study contrasting ‘spectral’ and ‘temporal’ modulations in noise-vocoded speech showed a greater response to spectral cues than temporal cues in the left STG [2].

In this study, we aimed to separately manipulate the amplitude and spectral modulations that occur in natural speech, which consist of a mix of modulation rates. One general difficulty in much work exploring speech-specific responses is the construction of adequate nonspeech analogues; that is to say, stimuli which have all the spectro-temporal complexity of speech (thus controlling for key acoustic properties) but which are not intelligible. Many nonspeech analogues have been used in the past, some of which are clearly inadequate as regards acoustic complexity (e.g., steady-state tones). On the other hand, it can be difficult to emulate the full spectro-temporal complexity of speech without making such signals partially intelligible. We have thus taken a different approach in which we simplify natural speech to contain only two kinds of modulations, which we know are necessary and sufficient for intelligibility. Such simplified stimuli are then much more readily modified to contain modulations with similar acoustic properties, but which are not intelligible.

In order to obtain modulations which we know are sufficient to support speech comprehension, we based our stimuli on so-called ‘sine-wave’ speech (SWS) [21] derived from simple sentences (e.g., *The wife helped her husband*). SWS consists of a small number of sinusoids whose frequency and amplitude are modulated to match the frequency and amplitude of the formants in speech. Although SWS is typically synthesized from three formant tracks, good intelligibility can be obtained from two formants only [21]. Furthermore, in order to provide spectral shapes more typical of natural speech, we noise-vocoded the stimuli [19] to broaden the single spectral components in SWS to more closely resemble formants. It may also be that this manipulation leads to more coherent and hence intelligible signals than that of SWS even though the information contained is identical, insofar as both signals contain spectral prominences varying in frequency and amplitude in the same way.

**Figure 1:**
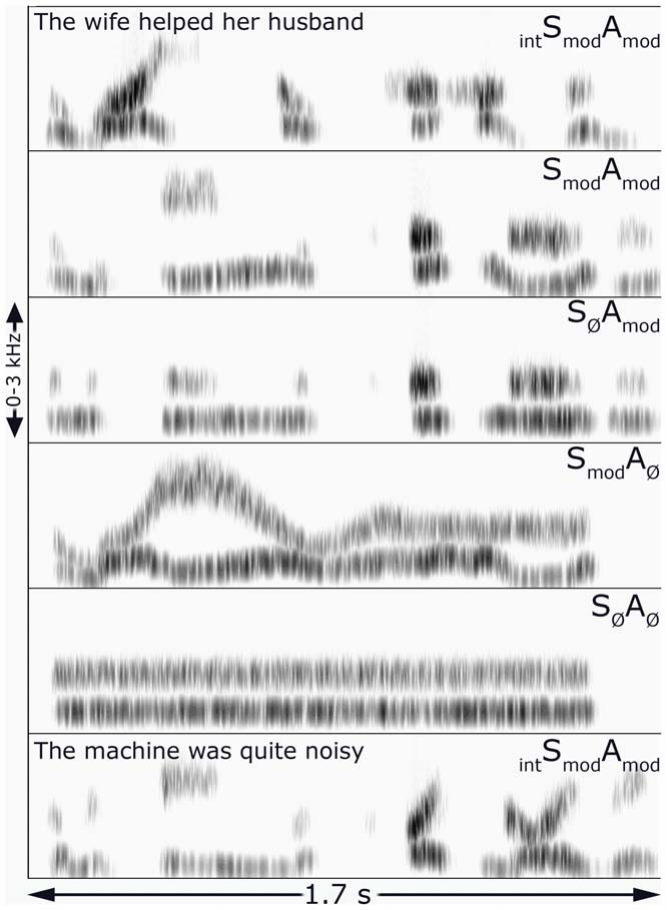
Representative spectrograms of the various stimulus conditions used. Time is on the x-axis, frequency on the y, with the darkness of the trace indicating the amount of energy present at each particular time/frequency co-ordinate. S = spectrum and A = amplitude, with the subscripted text indicating whether that feature is ***mod***ulated or not (**Ø** = no modulations). At top and bottom are the results of noise-vocoding sine wave representations of two natural sentences (tracking the frequency and amplitude of the lower two formants, spectral prominences arising primarily from the filtering action of the vocal tract). Such sounds are intelligible, as indicated by the prefix ***int.*** See the text for further details. [Audio S1 shows this figure along with audio examples.]

As Figure 1 [and Audio S1] show (top and bottom panels, labeled _int_S_mod_A_mod_), noise-vocoded SWS contains two spectral prominences which are modulated in both centre frequency (or **S**pectrum) and **A**mplitude across time. As both these sets of modulations come from a single sentence, this condition is *intelligible*, as indicated by the subscripted prefix. Across the four main conditions of our study, we selectively manipulated the presence or absence of each type of modulation. The simplest stimuli are S_Ø_A_Ø_ (the null subscript indicating a static feature), containing two spectral prominences which are modulated neither in spectrum nor amplitude. Two sets of stimuli are intermediate in acoustic complexity, varying only one type of modulation. S_mod_A_Ø_ stimuli do not vary in amplitude, but their spectral prominences are modulated by the formant frequencies extracted from the original sentence, interpolated over periods of zero amplitude. S_Ø_A_mod_ stimuli consist of fixed-frequency spectral prominences but with dynamic amplitude variation obtained from the original sine-wave characterization. Finally, the most complex stimuli are S_mod_A_mod_, in which both spectrum and amplitude are modulated, but with the two kinds of modulations taken from two different sentences. They are thus comparable in spectro-temporal complexity to _int_S_mod_A_mod_ stimuli, but are not intelligible.

Functional imaging was performed using PET while participants listened passively. There were two groups of participants: the first group heard only stimuli from the four classes of unintelligible noise-vocoded SWS, whereas the second group also heard the intelligible sounds as a fifth condition (being pre-trained on only these intelligible sounds before scanning). Apart from this training, none of the other test sounds was heard prior to scanning. Special care was taken not to imply that any of the unintelligible conditions were speech-like, but the second group did know that some of the stimuli would sound like their pre-training intelligible stimuli. After each scan every participant was asked the open ended question “what did those sounds sound like?”. A structured test (e.g. ratings of how speech- or voice-like the stimuli were) was not used, as we did not want to bias the subjects towards listening for particular properties in the stimuli. Every trial consisted of 64 different sentences in one particular condition, presented in a random order, with the stimulus presentation starting ∼15 seconds before the scan started. The order of conditions was randomized across the scanning sessions. There were seven participants in the untrained and six in the pre-trained condition, all native English speakers.

## Results

### Subjective reports

S_Ø_A_Ø_ stimuli were frequently described as sounding like ‘wind in the trees’ or ‘electronic vowel sounds’. S_Ø_A_mod_ stimuli were described as sounding ‘rhythmic’, ‘like a nursery rhyme’. S_mod_A_Ø_ stimuli were described as ‘sounding like speech with the bits taken out’, ‘like an alien’, ‘less rhythmic but more speech-like’, ‘a lunatic raving’. S_mod_A_mod_ stimuli were described as ‘very much like speech’, like people ‘with a regional accent’ or ‘aliens again’. Every one of the thirteen subjects commented that S_mod_A_mod_ stimuli sounded like speech but that they could not be understood. The six participants who had had pre-training and testing with _int_S_mod_A_mod_ stimuli all reported that these were fully intelligible.

### Brain areas sensitive to increasing acoustic complexity

**Figure 2:**
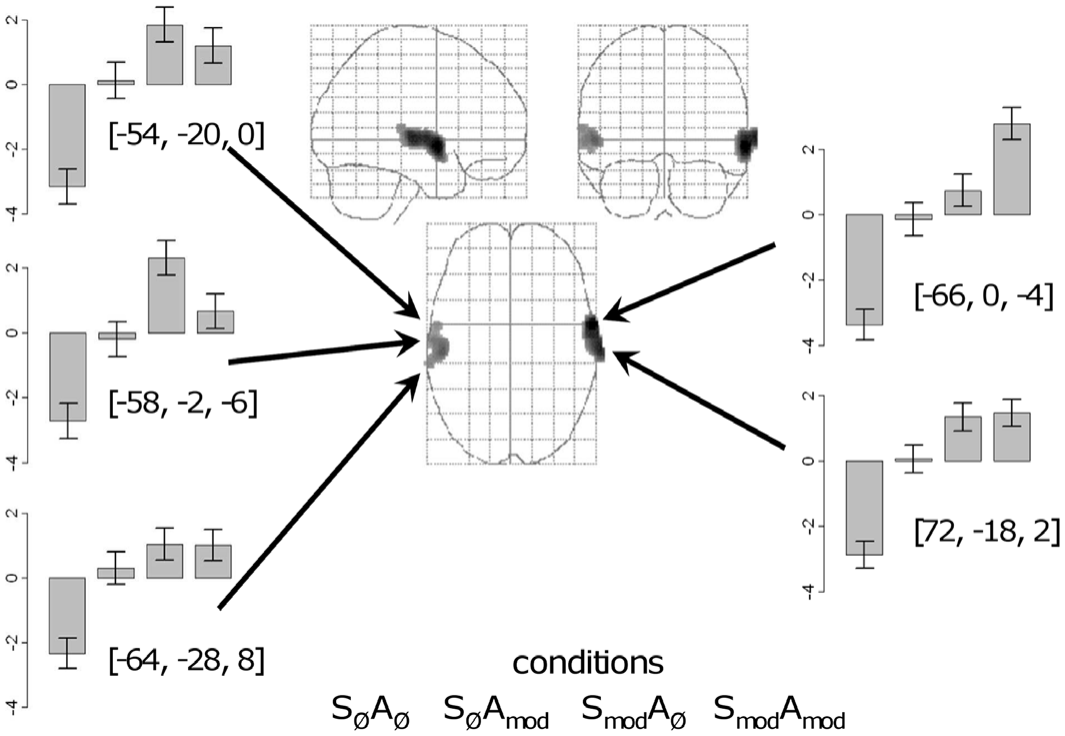
Neural activation revealed by the additive effects contrast S_mod_A_mod_>(S_mod_A_Ø_ and S_Ø_A_mod_)>S_Ø_A_Ø_. The activity, projected onto a “glass” brain, is thresholded at p<0.0001 with a cluster threshold of 40. The five activation plots show the mean effect sizes (centred on zero) with the error bars showing the standard error of the mean. The order of the conditions plotted is shown at the bottom of the figure.

The first contrast we investigated, over all 13 participants, concerned additive effects for modulation in unintelligible sounds only. Here we required the least activation for sounds with no modulation, increased and equal activation for both types of modulation on their own, and a further increase for simultaneous modulation of both features (a contrast of −1.0, 0.1, 0.1, +0.8 for conditions S_Ø_A_Ø_, S_mod_A_Ø_, S_Ø_A_mod_ and S_mod_A_mod_, respectively). As Figure 2 and Table 1 shows, this reveals bilateral activation, running lateral and anterior to primary auditory cortex (PAC), with a peak in the right anterior STG.

**Table 1:** Peak activations for the three contrasts investigated (using MNI co-ordinates).

Brain regions and extent	x	y	z	Z score
**Additive effects of modulations**				
Right anterior STG/STS (626 voxels)	**66**72	**0**−18	**−4**2	**7.36**6.31
Left STG (416 voxels)	**−54**−64−58	**−20**−28−2	**0**8−6	**5.05**4.344.10
**Effects of intelligible stimuli**				
Left posterior STS (251 voxels)	**−58**−68	**−22**−28	**0**0	**4.69**4.48
Left anterior STS (82 voxels)	**−52**−54	**8**14	**−20**−26	**4.68**3.42
Right anterior STG/STS (111 voxels)	**66**	**−2**	**−4**	**4.11**
**Effects of training with intelligible stimuli**				
pre SMA (42 voxels)	**0**−8	**10**10	**54**48	**4.07**3.33
Right DLPFC (45 voxels)	**38**38	**36**36	**32**46	**3.98**3.79

Bold values indicate the largest cluster in each region.

### Brain areas sensitive to intelligible speech

**Figure 3:**
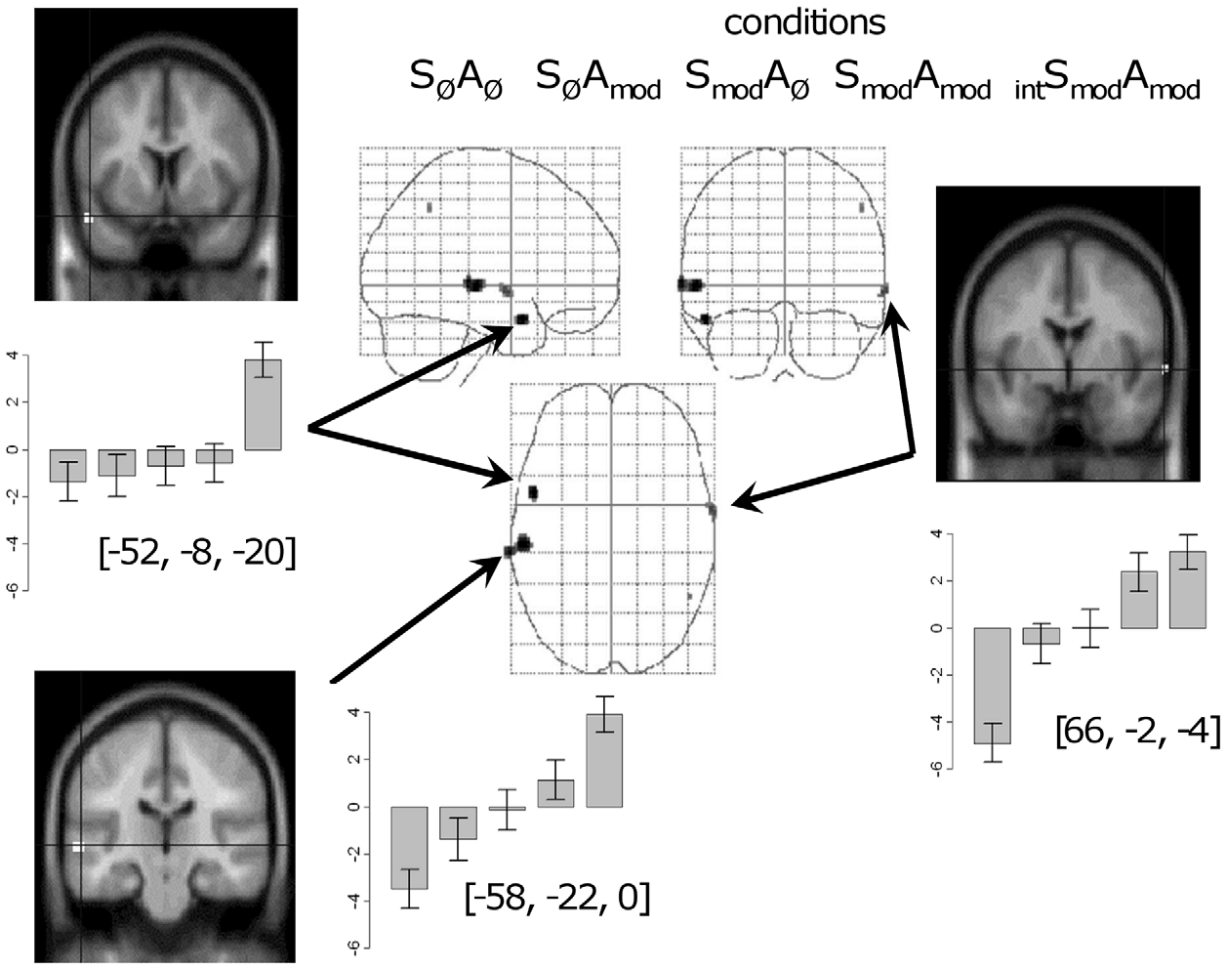
Neural activation revealed by the intelligibility contrast _int_S_mod_A_mod_>(S_mod_A_mod_, S_mod_A_Ø_ and S_Ø_A_mod_). This analysis is restricted to listeners who were pre-trained with the intelligible stimuli and who heard these stimuli in the scanner. The activation is projected onto a “glass” brain with the statistical constraints and error bars as described for Figure 2. Also shown are coronal sections pinpointing the regions for which activations as a function of condition have been plotted.

Within the six participants who heard _int_S_mod_A_mod_ stimuli in the PET scanner, we determined which brain regions showed increased activation for intelligible speech in comparison to all four unintelligible conditions (a contrast of −¼, −¼, −¼, −¼, +1 for conditions S_Ø_A_Ø_, S_mod_A_Ø_, S_Ø_A_mod_, S_mod_A_mod_, and _int_S_mod_A_mod_ respectively). As Figure 3 and Table 1 show, activation was bilateral, with peaks in the right and left superior temporal sulcus (STS). However, only in the left anterior STS, near the temporal pole, was the response solely to the intelligible _int_S_mod_A_mod_ condition, with no variation in activity with variation in acoustic features.

### Brain areas sensitive to the expectation of hearing intelligible stimuli

**Figure 4:**
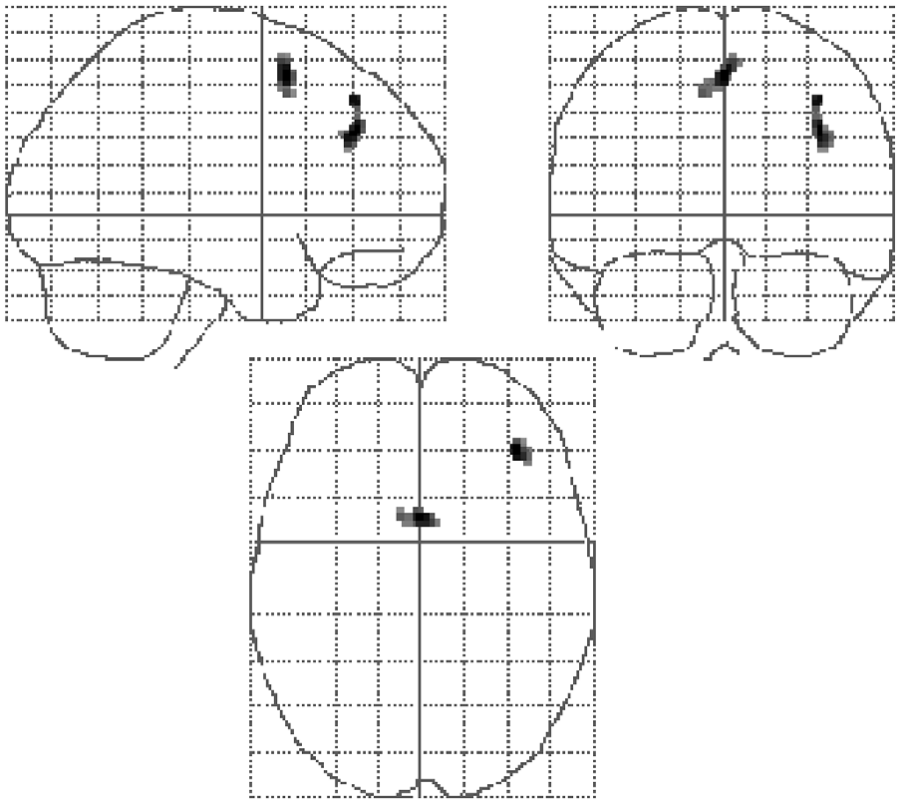
Differences in neural activation between the trained and naïve groups. Shown projected on to a “glass” brain are the activations which are greater in the trained as opposed to the naïve group of subjects for the additive effects contrast S_mod_A_mod_>(S_mod_A_Ø_ and S_Ø_A_mod_)>S_Ø_A_Ø_. The statistical constraints are as described for Figure 2.

Note that both groups of participants heard the complex acoustic condition S_mod_A_mod_. However, only the second group expected to hear intelligible stimuli during the scan, as a result of their being pre-trained with _int_S_mod_A_mod_ stimuli (which they also heard during the scan). A random effects comparison of the brain areas more activated in the pre-trained than the naïve participants for the additive response to the unintelligible S_mod_A_mod_ condition revealed significant activations only outside of the temporal lobes (Figure 4). These activations may reflect an increased effort to understand the stimuli, as other similar stimuli were, in fact, intelligible for the trained group. No brain areas were activated by the opposite contrast (i.e. naïve group>trained group).

## Discussion

Our results clearly show that there are cortical areas sensitive to the additive effects of spectral and amplitude modulations in speech, without this reflecting sensitivity to intelligibility. However, unlike the predictions of theories that hypothesise a specialized role for the left temporal lobe in the auditory (non-linguistic) processing of speech-related modulations in a signal, these additive effects are seen bilaterally, with the largest peak in the *right* anterior temporal lobe. A dominant response was seen in the left temporal lobe only when the stimuli not only had acoustic properties appropriate for speech, but were also intelligible. Indeed, in the left anterior STS, we found an area which responded to the intelligible _int_S_mod_A_mod_ condition, which was also insensitive to the differences among all four unintelligible conditions, even though they differed greatly in spectro-temporal complexity. This is the same area which we have previously demonstrated to respond selectively to intelligible speech compared to a different complex acoustically-matched baseline [22]. It therefore appears that crucial left temporal lobe systems involved in speech processing are not driven simply by low level acoustic features of the speech signal but require the presence of linguistic information to be activated.

That the left hemisphere dominance for speech is tied to linguistic, and not acoustic properties of the stimuli, has also been shown in a dichotic listening study in which speech sounds known as clicks are only left-lateralised in populations in whom these contrasts form a part of their native phonetic repertoire [23]. Such a result is also highly consistent with both behavioural [24] and physiological [25] evidence in non-human primates that left-lateralised neural processing is only recruited for conspecific vocalisations with communicative intent, and not to sounds with particular acoustic properties. Note, though, that at least one study has found broadly bilateral activations to conspecific vocalizations, and even a right-wards bias [26]. On the other hand, no previous study, primate or human, has so carefully matched the acoustic properties of the control stimuli to the conspecific vocalisations, so it can be difficult to be certain about exactly what aspects of the different types of stimuli lead to differential activations. It is also crucial to remember that no non-human communicative system approaches the complexity of human language, so it would not be surprising to find aspects of the neural representation of speech sounds to be different to those found in any other animal listening to its conspecific vocalisations.

Also interesting in this regard is that all participants commented that the S_mod_A_mod_ condition sounded like someone talking, although unintelligibly. However this speech-like (or perhaps voice-like) quality was not sufficient to result in a left dominant neural response. The dominant responses to the additive acoustic effects in the right STS may support arguments that voice processing is at least partly subserved by the right anterior STS, but that this does not depend on intelligibility [27].

We can also safely say that the neural responses in the additive effects contrast in the STS were not affected by underlying differences between the two groups of participants, one of which experienced intelligible stimuli both before scanning (during explicit training) and in the scanner. A direct comparison of this contrast in the two groups did show greater activation in the trained than the naïve group (i.e. more activation to stimuli on which they had not been pre-trained); however, this activation lay beyond the temporal lobes, in the pre-SMA and dorsolateral prefrontal cortex, regions which have been implicated in difficult listening conditions [28], subvocal articulation [29], and monitoring processes in speech [30].

All the participants in the pre-trained group thought that the S_mod_A_mod_ stimuli were the same as the _int_S_mod_A_mod_ stimuli, but that they could not quite understand the former. Perhaps they were recruiting these regions more than the naïve group because they had had experience developing articulatory strategies for dealing with this unfamiliar type of speech. However, despite their expectations and feeling that the S_mod_A_mod_ stimuli sounded like they should be understood, this did not lead to greater activation in the temporal lobes than the naïve participants had. It thus seems likely that the additive response to S_mod_A_mod_ seen in the right and left temporal lobes is driven by the acoustic properties of the stimuli, rather than deliberate attempts to understand them.

Overall, these findings indicate that left dominant temporal lobe responses to heard speech are not driven by low level acoustic properties of the stimuli. In contrast, there is some evidence that right dominant responses to voice-like stimuli, regardless of their intelligibility, *are* driven by low-level acoustic properties of the sounds, showing an additive response to amplitude and spectral modulations. It will be interesting to see how these effects can be related to the functional imaging work from humans revealing an auditory voice area which lies in the anterior right STG/STS [27]. It is possible that simple acoustic sensitivities may account for right temporal lobe dominance for voice-like stimuli, but not for left temporal lobe dominance for speech. In short, the differences between the processing exacted by the right and left auditory cortices cannot be adequately accounted for by a simple model where each processes opposing but complementary features in sound without regard for communicative function.

## Materials and Methods

### Participants

Thirteen right-handed native English-speaking volunteers (mean age of 43.7 years, ranging from 35–53, with 5 females) were recruited and scanned. None reported any hearing problems. Each participant gave informed consent prior to participation in the study, which was approved by the Research Ethics Committee of Imperial College School of Medicine/Hammersmith, Queen Charlotte's & Chelsea & Acton Hospitals. Permission to administer radioisotopes was given by the Department of Health (UK).

### Stimuli

All stimulus materials were based on digital recordings of simple everyday sentences from the BKB lists [31] made in an anechoic chamber by an adult male speaker of standard Southern British English (e.g., *The clown had a funny face*). A semi-automatic procedure (with extensive hand-checking and correcting) was first used to track the frequencies and amplitudes of up to three formants every 10 ms in 64 of these sentences. From these values were constructed sine-wave versions of the original speech [32], by synthesizing up to three independent sinusoids whose frequency and amplitude matched those of the original formants. The accuracy of the formant tracks was confirmed by the fact that this sine-wave speech led to relatively high levels of intelligibility in listeners after a very small amount of training (averaging about 87% of key words correct in a study with 16 young adults, where each sentence typically has three key words).

All further signal processing was done off-line, using special purpose programs written for MATLAB. All stimuli for this study were based on only the first two formant tracks. The essential structure of the stimulus manipulations was a 2×2 design, varying spectral complexity (formant frequencies modulated vs. formants static) and amplitude complexity (amplitude modulated vs. amplitude static). In order to provide formant tracks that varied continuously over the entire course of the utterance (through periods of silence, for example, due to consonantal closure) formant tracks were interpolated over these silent periods using piecewise-cubic Hermite interpolation in log frequency and linear time. Static formant tracks were set to the median frequencies of the measured formant tracks, separately for each formant track. Similarly, static amplitude values were obtained from the median of the measured amplitude values larger than zero.

The four manipulated stimulus conditions were thus (using the null symbol Ø to indicate a static feature for **S**pectrum or **A**mplitude):

- S_Ø_A_Ø_: Steady-state formant tracks at a fixed amplitude
- S_mod_A_Ø_: Dynamic interpolated formant tracks at a fixed amplitude
- S_Ø_A_mod_: Steady-state formant tracks with dynamic amplitude variation obtained from the original sine-wave characterization
- S_mod_A_mod_: Dynamic interpolated formant tracks from one utterance with the dynamic amplitude variations from another. Linear time scaling of the amplitude contours was performed as required to account for the different durations of the two utterances.

These will be referred to as the unintelligible conditions. For the intelligible stimuli (_int_S_mod_A_mod_), the sine-wave stimuli used the formant tracks and amplitudes from a single sentence, determined in a similar way, but with less extensive hand correction (the interpolation required for the unintelligible stimuli meant that small errors made even in periods of low signal intensity could make the interpolation unreliable). All stimuli were screened for intelligibility by the experimenters and those with audible errors or judged to be less intelligible than others (through informal listening) were discarded.

In order to make the stimuli sound less like bird calls, and to have spectral shaping similar to that experienced in speech (with broader formants in place of sinusoids), noise-excited vocoding [19] was applied in order to smear their spectra across frequency. Here, the input waveform was passed through a bank of 27 analysis filters (each a 6th-order Butterworth) with frequency responses that crossed 3 dB down from the pass-band peak. Envelope detection occurred at the output of each analysis filter by full-wave rectification and 2nd-order Butterworth low-pass filtering at 30 Hz. These envelopes were then multiplied by a white noise, and each filtered by a 6th-order Butterworth IIR output filter identical to the analysis filter. The rms level from each output filter was then set to be equal to the rms level of the original analysis outputs, before being summed together. Cross-over frequencies for both the filter bank (over the frequency range 70–5000 Hz) were calculated using an equation relating position on the basilar membrane to its best frequency [33]. Examples of the final stimuli can be seen in Figure 1.

The intelligibility of these stimuli (excluding the obviously unintelligible S_Ø_A_Ø_) was investigated in 13 young adult listeners who listened to 10 sentences from each of the conditions S_mod_A_Ø_, S_Ø_A_mod_ and S_mod_A_mod_, and 20 of _int_S_mod_A_mod_. Scoring by key words (from either sentence in the case of S_mod_A_mod_) led to a mean intelligibility score of 61%, 6%, 3% and 3% for conditions _int_S_mod_A_mod_, S_mod_A_mod_, S_mod_A_Ø_, and S_Ø_A_mod_ respectively. We also allowed for the possibility of intelligible speech arising by chance combinations by scoring as correct any words identified by three or more of the listeners, even if they were not in any of the constituent sentences. This increased overall intelligibility scores to 64%, 25%, 10% and 3%. To summarise, the intelligible condition leads listeners, on average, to reporting accurately almost 2 of the typical 3 key words, whereas the unintelligible conditions lead to a ‘correct’ percept less than once per sentence, even when listeners are urged to report meaningful words.

### PET scanning and procedure

PET scanning was performed with a Siemens HR++ (966) PET scanner operated in high-sensitivity 3D mode. Sixteen scans were performed on each participant, using the oxygen-15-labelled water bolus technique. All participants were scanned whilst lying supine in a darkened room with their eyes closed.

The stimuli were presented at a comfortable level determined for each participant, kept constant over the scanning sessions. The sentence presentations began 15 seconds before the scanning commenced, and no sentence was repeated within a condition. The participants were instructed to listen passively to the sounds. At the end of each scanning trial, every participant was asked what they stimuli had sounded like to them.

All participants heard the 4 unintelligible conditions. None of these conditions were referred to by the experimenters as speech-like in any way. Seven of the participants heard only these 4 conditions, 4 times each. Six participants were pre-trained on intelligible stimuli (_int_S_mod_A_mod_,) which were included as an extra scanning condition. They heard the 4 unintelligible conditions 3 times each, and the intelligible condition 4 times. All conditions were presented in a random order different for each participant.

### Analysis

Images were analysed using SPM99 software on grouped data (http://www.fil.ion.ucl.ac.uk/spm/software/spm99/). All scans from each participant were realigned to eliminate head movements between scans and normalised into a standard stereotactic space. Images were then smoothed using an isotropic 10 mm, full width half-maximum, Gaussian kernel, to allow for variation in gyral anatomy and to improve the signal-to-noise ratio. Specific effects were investigated, voxel-by-voxel, using appropriate contrasts to create statistical parametric maps of the t statistic, which were subsequently transformed into Z scores. The analysis included a blocked AnCova with global counts as confound to remove the effect of global changes in perfusion across scans. The threshold for significance was set at *p*<0.05, corrected for analyses across the whole volume of the brain (*p*<0.000001, uncorrected; Z-score>4.7).

## Supporting Information

Audio S1 **Representative spectrograms and sounds for the various stimulus conditions.** For each row of the figure, time is on the x-axis, frequency on the y, with the darkness of the trace indicating the amount of energy present at each particular time/frequency co-ordinate. Each row gives a single example from a particular condition. Conditions are named using the indicators S = spectrum and A = amplitude, with the subscripted text indicating whether that feature is **mod**ulated or not (**Ø** = no modulations). The prefix **int** indicates a condition that is intelligible as both spectrum and amplitude are modulated with features derived from the same sentence. The loudspeaker icons on the right will play the sentence in the specified condition when pressed. Icons on the left, at top and bottom, play the original audio of the sentences from which these examples were constructed. See the text for further details.
(PPT)
