# Supplementary material for: Hemispheric Asymmetries in Speech Perception: Sense, Nonsense and Modulations
Source: PLoS One. 2011 Sep 30;6(9):e24672. doi: 10.1371/journal.pone.0024672 (PMC3184092; doi:10.1371/journal.pone.0024672)
Supplement: Audio S1 — Representative spectrograms and sounds for the various stimulus conditions. For each row of the figure, time is on the x-axis, frequency on the y, with the darkness of the trace indicating the amount of energy present at each particular time/frequency co-ordinate. Each row gives a single example from a particular condition. Conditions are named using the indicators S = spectrum and A = amplitude, with the subscripted text indicating whether that feature is modulated or not (Ø = no modulations). The prefix int indicates a condition that is intelligible as both spectrum and amplitude are modulated with features derived from the same sentence. The loudspeaker icons on the right will play the sentence in the specified condition when pressed. Icons on the left, at top and bottom, play the original audio of the sentences from which these examples were constructed. See the text for further details. (PPT) [file pone.0024672.s001.ppt]

## Slide 1
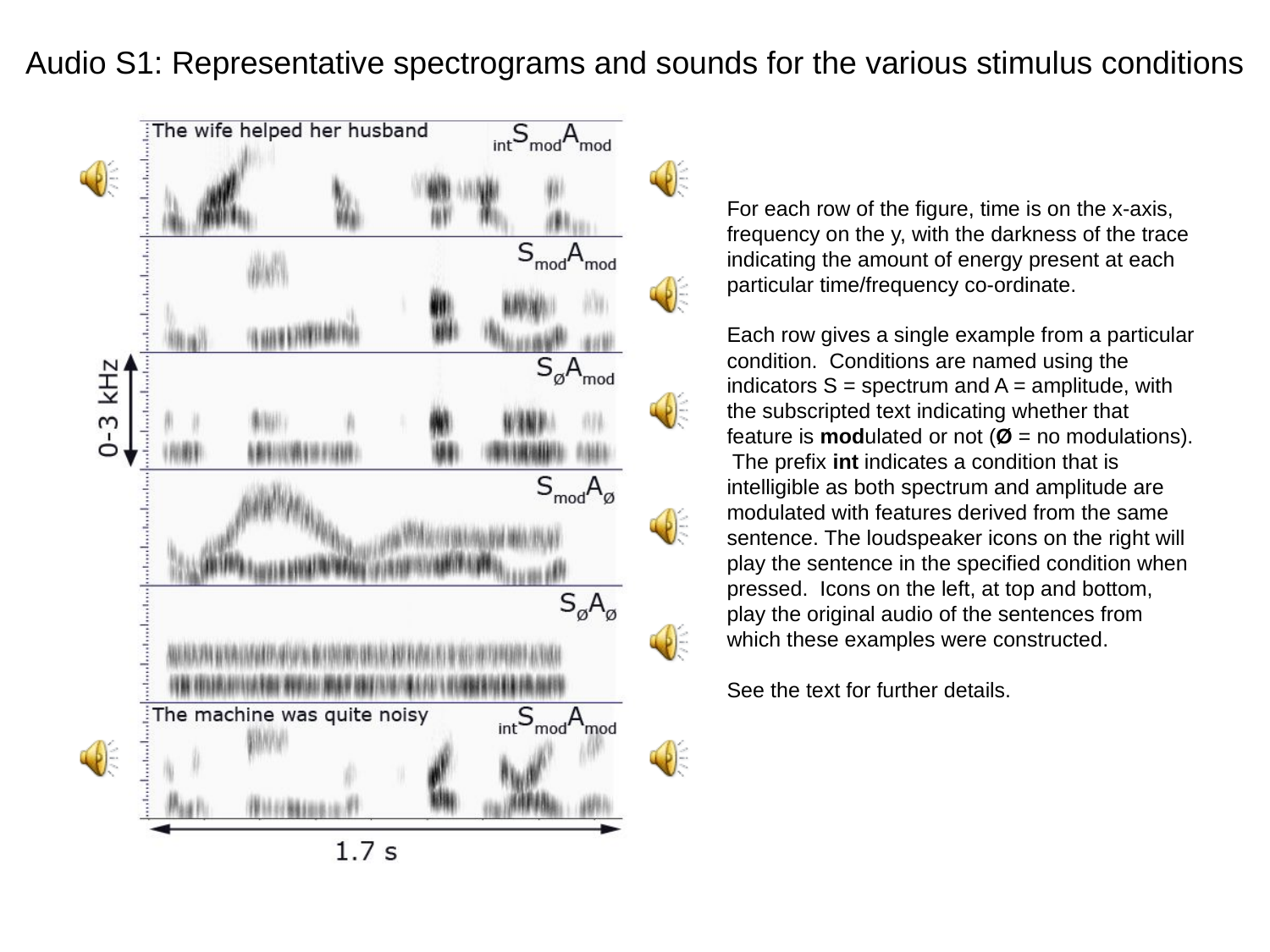

Audio S1: Representative spectrograms and sounds for the various stimulus conditions
For each row of the figure, time is on the x-axis, frequency on the y, with the darkness of the trace indicating the amount of energy present at each particular time/frequency co-ordinate.
Each row gives a single example from a particular condition. Conditions are named using the indicators S = spectrum and A = amplitude, with the subscripted text indicating whether that feature is modulated or not (Ø = no modulations). The prefix int indicates a condition that is intelligible as both spectrum and amplitude are modulated with features derived from the same sentence. The loudspeaker icons on the right will play the sentence in the specified condition when pressed. Icons on the left, at top and bottom, play the original audio of the sentences from which these examples were constructed.
See the text for further details.
